# Physicochemical and Multimodal Imaging Properties of Core–Shell Ln_2_O_3_@Carbon Nanoparticles (Ln = Tb and Ho)

**DOI:** 10.3390/molecules30204064

**Published:** 2025-10-12

**Authors:** Huan Yue, Tirusew Tegafaw, Shuwen Liu, Ying Liu, Dejun Zhao, Endale Mulugeta, Xiaoran Chen, Ahrum Baek, Kwon Seok Chae, Jihyun Kim, Yongmin Chang, Gang Ho Lee

**Affiliations:** 1 Department of Chemistry, College of Natural Sciences, Kyungpook National University, Taegu 41566, Republic of Koreatirukorea@gmail.com (T.T.); djzhao.chem@gmail.com (D.Z.);; 2 Institute of Biomedical Engineering Research, School of Medicine, Kyungpook National University, Taegu 41944, Republic of Korea; 3 Department of Biology Education, Teachers’ College, Kyungpook National University, Taegu 41566, Republic of Korea; 4 Department of Chemistry Education, Teachers’ College, Kyungpook National University, Taegu 41566, Republic of Korea; jkim23@knu.ac.kr; 5 Department of Molecular Medicine, School of Medicine, Kyungpook National University, Taegu 41944, Republic of Korea

**Keywords:** core-shell nanoparticle, Ln_2_O_3_@carbon (Ln = Tb and Ho), cellular toxicity, relaxivity, T_2_ MRI contrast agent, photoluminescence

## Abstract

In this study, core–shell Ln_2_O_3_@carbon nanoparticles (core = Ln_2_O_3_ and shell = carbon; Ln = Tb and Ho) were synthesized for the first time by preparing Ln_2_O_3_ nanoparticles through a polyol method, followed by carbon coating using D-glucose as a carbon precursor in aqueous media. The synthesized Ln_2_O_3_@carbon nanoparticles exhibited good colloidal stability in solution and very low toxicity in in vitro cellular cytotoxicity tests. They exhibited paramagnetic magnetization values that increased with increasing applied field strength, resulting from spin–orbit magnetic moments of 4f-electrons; hence, they yielded negligible r_1_ (<0.1 s^−1^mM^−1^) and appreciable r_2_ values (3.446 and 3.677 s^−1^mM^−1^ for Ln = Tb and Ho, respectively) at 3 T, highlighting their potential as T_2_ MRI contrast agents, particularly at high MR fields. In addition, the carbon coating shell exhibited photoluminescence at 460 nm, suitable for applications in fluorescence imaging probes.

## 1. Introduction

Nanomedicine, i.e., nanotechnology-based medicine, has attracted great attention owing to its strong potential to overcome the limitations of conventional medicine, thus representing a breakthrough in disease treatments [1,2,3]. Among various examples, nanoparticle-based contrast agents for magnetic resonance imaging (MRI) are emerging as potential alternatives to the conventional molecular imaging contrast agents employed in MRI, such as Gd(III)-chelates [4,5,6], because they can provide enhanced imaging performances, reduced dosages, longer blood circulation times, and targeted theranosis [7,8,9].

The MRI contrast agents function by enhancing the proton spin relaxation rates at their accumulation region, leading to contrast enhancement [4,5,6,10]. Various types of nanoparticle-based negative (T_2_) MRI contrasts have been introduced to date [11,12,13,14,15,16,17,18,19]; however, large nanoparticles [diameter (d) > 5 nm], such as superparamagnetic iron oxide nanoparticles (SPIONs), tend to exhibit long-term accumulation in the body, thus potentially causing biotoxic effects such as back pain [20,21,22]. Another system suitable for use as T_2_ MRI contrast agents is lanthanide (Ln)-based nanoparticles [12]. However, ultrasmall nanoparticles (d < 3 nm) are extremely useful as T_2_ MRI contrast agents owing to their renal excretion ability [23,24,25]. Lanthanide (Ln)-based nanoparticles are ideal systems for this purpose, because their magnetic properties are nearly independent of the size and the surface-coating ligand [26,27], enabling their fabrication in ultrasmall sizes and their surface to be coated with any kind of ligand. The most effective T_2_ relaxation results from electron spin–orbit magnetic moments of fast moving 4f electrons, because they can exclusively induce T_2_ relaxation along with minimal T_1_ relaxation [28,29], in contrast to pure electron spin magnetic moments of slow moving 3d or 4f electrons, which induce strong T_1_ relaxations [28,30,31,32]. Therefore, ultrasmall Ln_2_O_3_ nanoparticles (Ln = Tb and Ho) are ideal systems to serve as highly effective T_2_ MRI contrast agents, owing to the high paramagnetic electron spin–orbit moments of Tb and Ho, resulting from their 4f electrons [12,29,33] and renal excretion ability [23,24,25].

In this study, carbon-coated ultrasmall Ln_2_O_3_ nanoparticles (i.e., core–shell Ln_2_O_3_@carbon; Ln = Tb and Ho) were synthesized for the first time to demonstrate their potential as T_2_ MRI contrast agents. Ultrasmall Ln_2_O_3_ nanoparticles were synthesized via a polyol method and then coated with carbon by reducing D-glucose with NaOH in the presence of the nanoparticles in aqueous media. Carbon, as a key element in living organisms, is nontoxic and thus suitable for use as a surface-coating material for biomedical applications [34,35,36,37]. In addition, as confirmed in this study, carbon nanomaterials emit photons in the visible region [38,39,40], which makes them useful as fluorescent imaging probes. To investigate their potential as T_2_ MRI contrast agents, we assessed various physicochemical properties, including in vitro cellular toxicities and water proton spin relaxivities.

## 2. Results

### 2.1. Physicochemical Properties

#### 2.1.1. Particle Size

**Figure 1:**
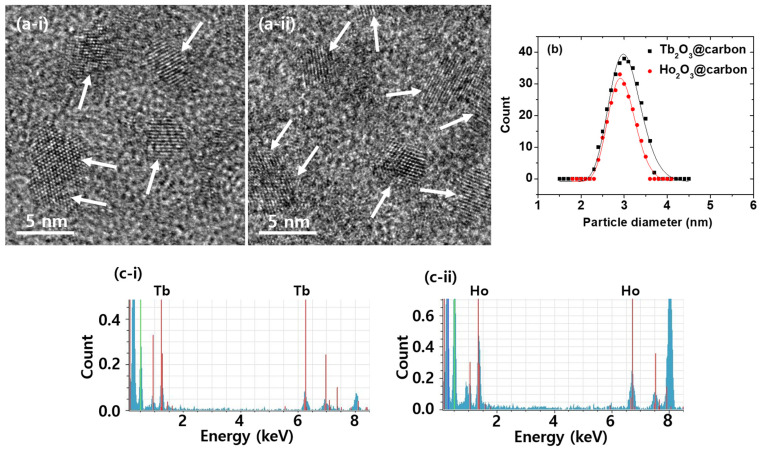
(**a**) HRTEM images of core–shell Ln_2_O_3_@carbon nanoparticles. Arrows indicate nanoparticles. (**b**) Log-normal function fits to observed particle diameter distributions. (**c**) EDS spectra. Ln = (**i**) Tb and (**ii**) Ho in (**a**,**c**).

To confirm the successful synthesis of ultrasmall core–shell Ln_2_O_3_@carbon nanoparticles (Ln = Tb and Ho), the synthesized materials were characterized using various techniques. As shown by high-resolution transmission electron microscopy (HRTEM) images (Figure 1a), the Ln_2_O_3_@carbon nanoparticles had particle diameters ranging from 2 to 4 nm, with average values (d_avg_) of 3.0 and 2.9 nm for Ln = Tb and Ho, respectively (Table 1), as estimated using log-normal function fits to the observed particle diameter distributions (Figure 1b). Clear lattice fringes were observed, confirming the successful synthesis of the Ln_2_O_3_ nanoparticles. In addition, energy dispersive X-ray spectroscopy (EDS) exhibited the presence of Tb and Ho in the nanoparticles (Figure 1c).

#### 2.1.2. Hydrodynamic Diameter

**Figure 2:**
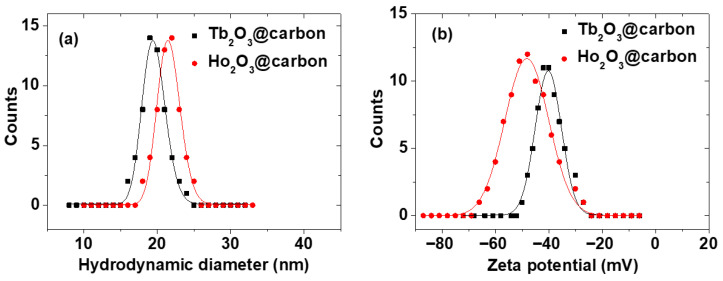
(**a**) DLS patterns and log-normal function fits to estimate a_avg_ values and (**b**) zeta potential curves of core–shell Ln_2_O_3_@carbon nanoparticles (Ln = Tb and Ho) dispersed in triple-distilled water.

The average hydrodynamic diameter (a_avg_) of the Ln_2_O_3_@carbon nanoparticles was estimated to be 19.5 and 21.5 nm for Ln = Tb and Ho, respectively, from log-normal function fits to the observed dynamic light scattering (DLS) patterns (Figure 2a; Table 1). The larger a_avg_ than d_avg_ values are due to the carbon coating and water hydration layer around the nanoparticles, because the surface functional groups of the carbon shells are -OH groups originating from D-glucose, used as a carbon precursor. This was supported by the highly negative zeta potentials (ζ) of the core–shell nanoparticles, i.e., −48.2 and −40.2 mV for Ln = Tb and Ho, respectively (Figure 2b; Table 1).

#### 2.1.3. Colloidal Stability

**Figure 3:**
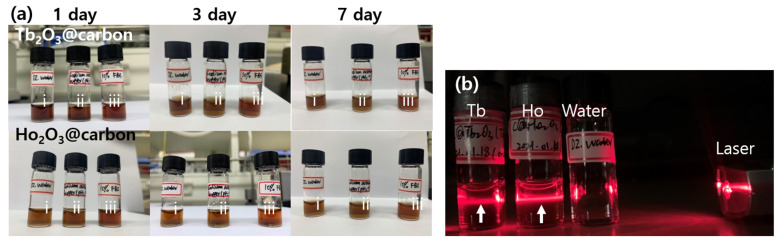
(**a**) Photographs of solutions containing core–shell Ln_2_O_3_@carbon nanoparticles (Ln = Tb and Ho) in (i) triple-distilled water, (ii) sodium acetate buffer (pH = 7), and (iii) 10% FBS solutions at 1, 3, and 7 days. (**b**) Laser scattering (or Tyndall effect, indicated with vertical arrows, measured using a commercial laser pointer with 650 nm wavelength) of triple-distilled water (used as reference) and core–shell Ln_2_O_3_@carbon nanoparticles (Ln = Tb and Ho) dispersed in triple-distilled water.

The physicochemical properties and performance of nanoparticles may be affected by colloidal stability, and thus good colloidal stability is crucial for biomedical applications [41]. The colloidal stability of the ultrasmall core–shell Ln_2_O_3_@carbon nanoparticles (Ln = Tb and Ho) was investigated in triple-distilled water, sodium acetate buffer (pH = 7), and 10% fetal bovine serum (FBS) solutions at 1, 3, and 7 days (Figure 3a). As shown in Figure 3a, all solution samples were transparent, with no nanoparticle precipitation for up to 7 days, demonstrating their good colloidal stability. Moreover, Figure 3b shows that all nanoparticle solution samples except triple-distilled water exhibited laser scattering (i.e., Tyndall effect), confirming the good colloidal dispersion of the carbon-coated nanoparticles in triple-distilled water.

#### 2.1.4. Crystallinity

**Figure 4:**
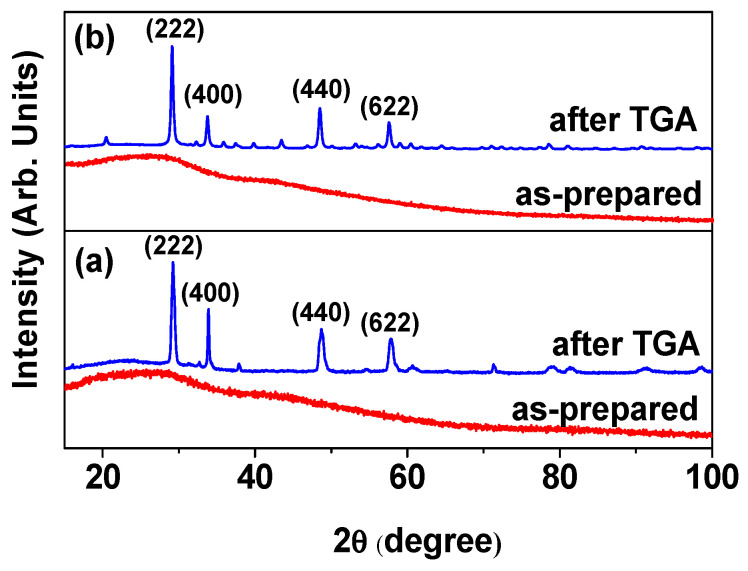
XRD patterns of core–shell Ln_2_O_3_@carbon nanoparticles before (**bottom**) and after (**top**) TGA: Ln = (**a**) Tb and (**b**) Ho. All peaks after TGA could be assigned with (hkl) Miller indices of cubic structure (card no. 00-021-1208 and 01-083-0923 for Tb and Ho, respectively), but only four strong peaks were assigned.

As shown by the X-ray diffraction (XRD) patterns in Figure 4a,b, the ultrasmall core–shell Ln_2_O_3_@carbon nanoparticles (Ln = Tb and Ho) exhibited very broad peaks (bottom patterns), indicating an amorphous structure due to their ultrasmall particle sizes; on the other hand, a cubic phase (top patterns) was observed after thermalgravimetric analysis (TGA), with lattice constants (a) of 10.57 and 10.61 Å for Ln = Tb and Ho, respectively, using Bragg’s equation, consistent with the literature values (10.72 and 10.6186 Å for Ln = Tb and Ho, respectively) [42,43].

#### 2.1.5. Carbon Coating Results

**Figure 5:**
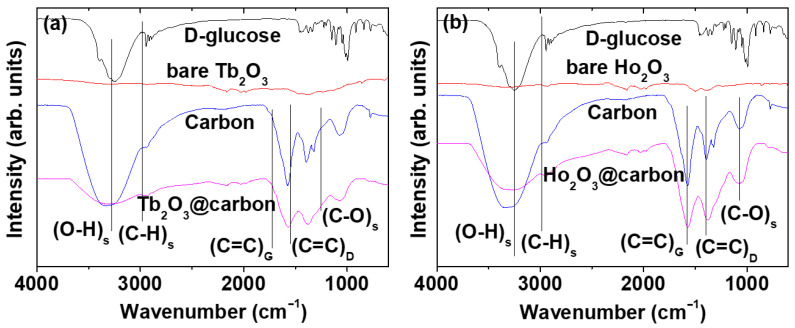
FT-IR absorption spectra of core–shell Ln_2_O_3_@carbon nanoparticles, D-glucose, bare Ln_2_O_3_ nanoparticles, and carbon nanoparticles (used as references): Ln = (**a**) Tb and (**b**) Ho. Subscripts s, G, D indicate “stretch”, “G-band”, and “D-band”, respectively.

**Figure 6:**
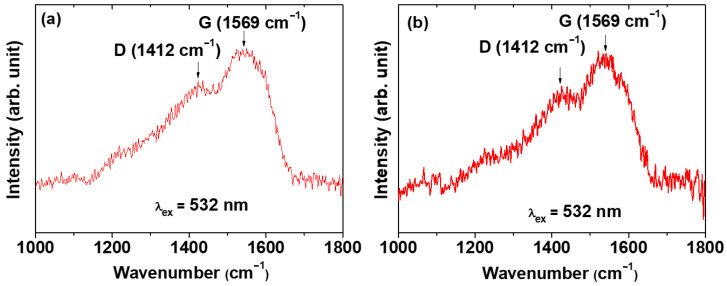
Raman spectra (λ_ex_ = 532 nm) of core–shell Ln_2_O_3_@carbon nanoparticles: Ln = (**a**) Tb and (**b**) Ho.

The carbon coating of the core–shell Ln_2_O_3_@carbon nanoparticles was analyzed through Fourier transform-infrared (FT-IR) absorption spectroscopy, Raman spectroscopy, elemental analysis (EA), and TGA. The FT-IR absorption spectra of nanoparticle samples and reference materials (i.e., D-glucose, carbon nanoparticles made from D-glucose, bare Ln_2_O_3_ nanoparticles after TGA) are displayed in Figure 5a,b for Ln = Tb and Ho, respectively. The C=C stretching-related G and D bands at 1577 and 1383 cm^−1^ [44,45], respectively, which also appeared in the FT-IR spectrum of the carbon nanoparticles, confirmed the presence of the carbon coating shell on the Ln_2_O_3_ nanoparticle surface. The strong O–H and C–O stretching peaks at 3252 and 1053 cm^−1^, respectively, indicated the presence of a large amount of −OH groups on the carbon coating shell, because D-glucose was used as the carbon source. The C–H stretching peak at 2943 cm^−1^ also confirmed the presence of the carbon coating on the nanoparticle surface. The G and D bands also appeared at 1569 and 1412 cm^−1^, respectively, in the Raman spectra (λ_ex_ = 532 nm) shown in Figure 6a (Ln = Tb) and Figure 6b (Ln = Ho), further confirming the presence of the carbon coating on the nanoparticle surface [45,46].

**Figure 7:**
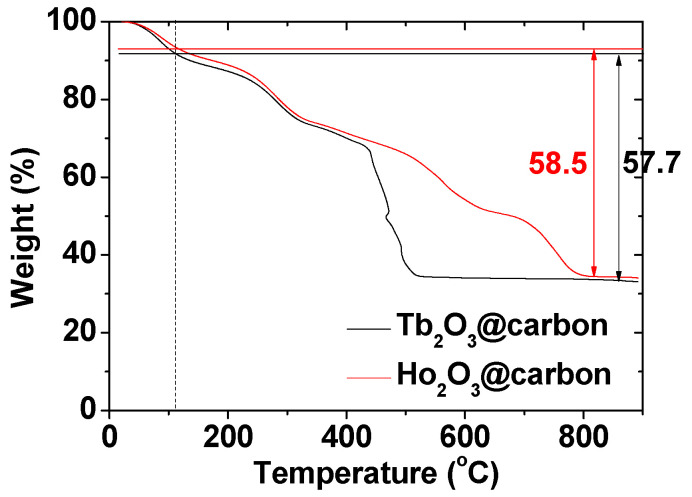
TGA curves of core–shell Ln_2_O_3_@carbon nanoparticles (Ln = Tb and Ho). Air and water desorption occurred before the vertical dotted line at ~110 °C and after it, the removal of carbon coating from the nanoparticle surface occurred.

The carbon coating amount on the nanoparticle surface was estimated from the TGA curves (Figure 7). The initial mass losses of 9.0 and 7.2 wt.% were due to water and air desorption from the powder samples of the core–shell Ln_2_O_3_@carbon nanoparticles (Ln = Tb and Ho). The subsequent mass losses, estimated as 57.7 and 58.5 wt.% for Ln = Tb and Ho, respectively, were due to the removal of carbon coating from the nanoparticle surface through an oxidation reaction with flowing hot air (Table 1). The remaining masses of 33.3 and 34.3 wt.% after TGA were due to the Ln_2_O_3_ nanoparticles (Ln = Tb and Ho, respectively), as identified from their XRD patterns (top patterns in Figure 4a,b).

The EA data provided in Table 1 show carbon coating amounts of 59.5 and 61.8 wt.% for core–shell Ln_2_O_3_@carbon nanoparticles (Ln = Tb and Ho, respectively), consistent with the TGA data. In addition, compared to the D-glucose (C_6_H_12_O_6_, C:H:O = 1:2:1), the estimated C:H:O ratios were 1:1.3:0.6 for Ln = both Tb and Ho; this indicated the presence of a large amount of H and O in carbon coating shells, consistent with the observation of C–H and O–H stretching peaks in the FT-IR absorption spectra.

### 2.2. Magnetic Properties

**Figure 8:**
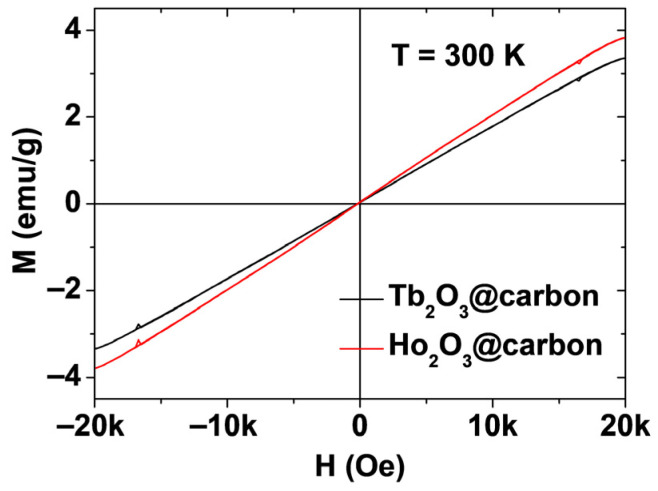
M–H curves of core–shell Ln_2_O_3_@carbon nanoparticles (Ln = Tb and Ho) at T = 300 K. The plots show the net M values of the core nanoparticles only.

The magnetic properties of powder samples of the core–shell Ln_2_O_3_@carbon nanoparticles (Ln = Tb and Ho) were characterized by measuring their magnetization versus applied magnetic field (M–H) curves at T = 300 K. The measured M values were corrected using the net masses of the Ln_2_O_3_ nanoparticles only (without carbon coating), which were estimated from the TGA curves. The mass-corrected net M values were used in the plots shown in Figure 8. The net M values were appreciable at room temperature and increased linearly with increasing H, reaching values of 3.37 and 3.82 emu/g at room temperature and H = 2 T (Table 1). These magnetization values are approximately ten times higher than 0.37 emu/g of carbon nanoparticles [47]. Therefore, the contribution of surface-coating carbon shells to magnetic moments of the nanoparticles is negligible. The magnetic moment of Ho_2_O_3_@carbon nanoparticles was slightly higher than that of Tb_2_O_3_@carbon nanoparticles because of a slightly higher magnetic moment of Ho^3+^ (^5^I_8_) compared to Tb^3+^ (^7^F_6_) [48]. This behavior confirms the paramagnetic nature of the Ln_2_O_3_@carbon nanoparticles, similar to the bulk [49,50,51,52], suggesting that they will induce stronger transverse (T_2_) water proton spin relaxations with increasing H.

### 2.3. In Vitro Cellular Cytotoxicity

**Figure 9:**
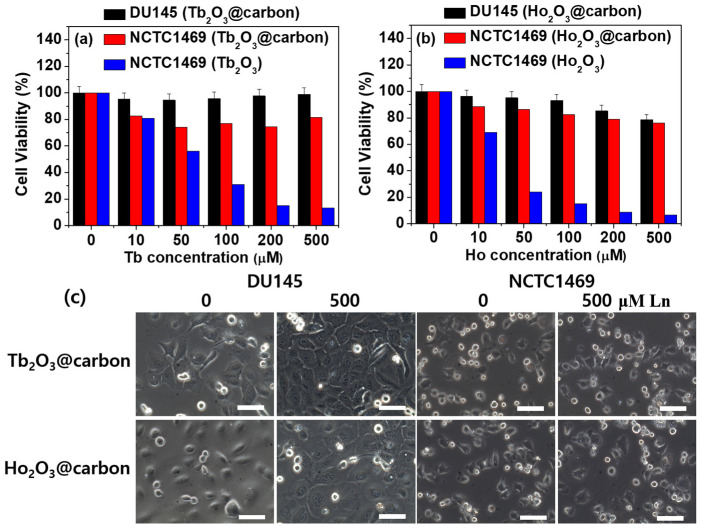
In vitro cell viabilities of core–shell Ln_2_O_3_@carbon nanoparticles in NCTC1469 and DU145 cells: Ln = (**a**) Tb and (**b**) Ho. (**c**) Optical microscope images of NCTC1469 and DU145 cells after treatment with 0 (control) and 500 μM Ln nanoparticle solutions (Ln = Tb and Ho) after 48 h. Scale bars indicate 70 μm.

The in vitro cell viability of the core–shell Ln_2_O_3_@carbon nanoparticles (Ln = Tb and Ho) was assessed using NCTC1469 and DU145 cell lines, and the results were compared to those obtained for the bare Ln_2_O_3_ nanoparticles. As shown in Figure 9a,b for Ln = Tb and Ho, respectively, the nanoparticles exhibited low cellular toxicity up to a measured Ln concentration of 500 μM after 48 h incubation, whereas the bare nanoparticles showed high toxicity, demonstrating the importance of the carbon coating in order to ensure a low toxicity of the nanoparticles. As shown in Figure 9c, the optical microscope images of the cells treated with 500 μM Ln (Ln = Tb and Ho) nanoparticle solutions after 48 h of incubation were similar to those of control cells (i.e., labeled as 0, untreated cells with nanoparticle solutions), confirming the low toxicity of the carbon-coated nanoparticles.

### 2.4. Water Proton Spin Relaxivities

**Figure 10:**
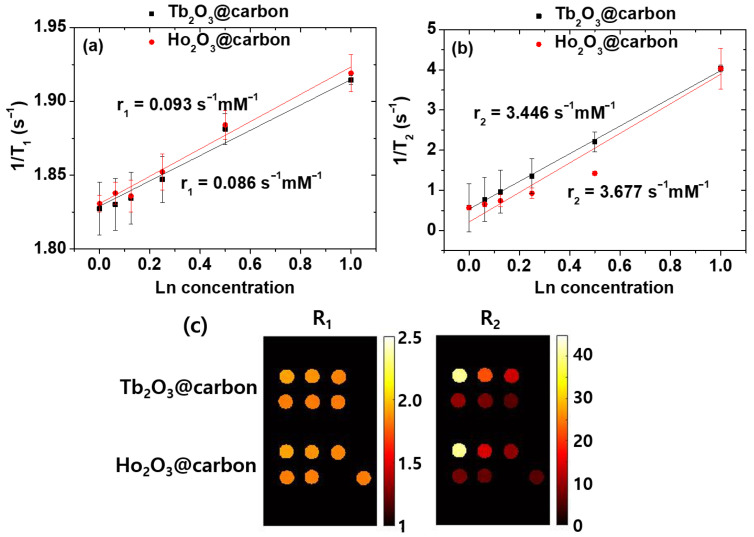
Plots of inverse (**a**) 1/T_1_ and (**b**) 1/T_2_ water proton spin relaxation times, along with (**c**) R_1_ and R_2_ map images of core–shell Ln_2_O_3_@carbon nanoparticles (Ln = Tb and Ho) dispersed in triple-distilled water as a function of Ln concentration.

As shown in Figure 10a,b, the inverse 1/T_1_ and 1/T_2_ water proton spin relaxation times of the core–shell Ln_2_O_3_@carbon nanoparticles (Ln = Tb and Ho) dispersed in triple-distilled water were plotted as a function of the Ln concentration, and the corresponding longitudinal (r_1_) and transverse (r_2_) relaxivity values were estimated from the corresponding slopes, respectively. The r_1_ values were negligible, i.e., 0.086 and 0.093 s^−1^mM^−1^ for Ln = Tb and Ho, respectively, whereas the r_2_ values were appreciable, i.e., 3.446 and 3.677 s^−1^mM^−1^ for Ln = Tb and Ho, respectively (Table 1). The negligible r_1_ values are attributed to the spin–orbit magnetic moments of fast moving 4f-electrons of Tb and Ho [28,48], while the appreciable r_2_ values are due to the appreciable magnetic moments of the nanoparticles [53,54], as observed in the M–H curves in Figure 8. However, the r_2_ values will increase with increasing H, because the paramagnetic M values of the nanoparticles increase with H, as shown in Figure 8.

As shown in Figure 10c, the R_1_ maps of the nanoparticles exhibited negligible dose-dependent contrast changes, whereas appreciable changes were observed in the R_2_ map images, demonstrating the ability of the nanoparticles to induce negative contrasts in vitro and thus their potential to serve as T_2_ MRI contrast agents.

### 2.5. Fluorescent Properties

**Figure 11:**
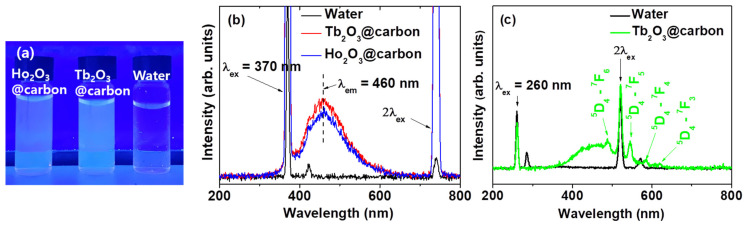
(**a**) Photographs under 365 nm UV irradiation and (**b**) PL spectra of core–shell Ln_2_O_3_@carbon nanoparticles (Ln = Tb and Ho) (λ_ex_ = 370 nm) and (**c**) core–shell Tb_2_O_3_@carbon nanoparticles (λ_ex_ = 260 nm) dispersed in triple-distilled water. Water was used as reference.

The carbon nanomaterials have unique photofluorescence properties in the visible region, making them suitable as fluorescent imaging probes [55,56,57]. The core–shell Ln_2_O_3_@carbon nanoparticles (Ln = Tb and Ho) dispersed in triple-distilled water exhibited photoluminescence (PL) under 365 nm UV irradiation, which is close to the excitation wavelength of the surface-coating carbon shells (available from a commercial UV lamp, VL-6.LC, Vilber Lourmat, Collégien, France), displaying a sky-blue emission color originating from the surface-coating carbon shells [47] (Figure 11a). As shown in Figure 11b, the nanoparticles exhibited PL spectra between 410 and 560 nm, originating from the surface-coating carbon shells [47], with emission maxima (λ_em_) at 460 nm at an excitation wavelength (λ_ex_) of 370 nm, demonstrating their potential as fluorescent imaging probes. This visible emission is suitable for in vivo skin and in vitro cellular imaging owing to its limited penetration depth. In addition, we observed emission peaks from the core Tb_2_O_3_ nanoparticles at λ_ex_ of 260 nm (Figure 11c), indicating that both the core and the carbon-coating shell in core–shell Tb_2_O_3_@carbon nanoparticles will be useful for fluorescent imaging.

## 3. Discussion

In this study, ultrasmall core–shell Ln_2_O_3_@carbon nanoparticles (Ln = Tb and Ho) were successfully synthesized, as confirmed via various analytical techniques. The carbon coating shell endowed the nanoparticles with multifunctional properties, i.e., good colloidal stability, reduced toxicity, and photoluminescence in the visible region centered at 460 nm. Because the carbon coating shell was synthesized using D-glucose as a carbon source, it contained numerous hydrophilic −OH groups, as confirmed by FT-IR absorption spectroscopy (Figure 5a,b) and EA (discussed at the last paragraph in Section 2.1.5), thus yielding highly negative zeta potentials (Figure 2b) and good colloidal stability in solution (Figure 3a). Moreover, the core–shell Ln_2_O_3_@carbon nanoparticles exhibited almost complete nontoxicity in in vitro cellular toxicity tests using NCTC1469 and DU145 cells, whereas the bare Ln_2_O_3_ nanoparticles were toxic (Figure 9a,b). This is because carbon, as one of the most common elements in living organisms, is nontoxic [34,35,36,37] and can thus be used as nanoparticle coating materials.

The paramagnetic moments of the core–shell Ln_2_O_3_@carbon nanoparticles (Ln = Tb and Ho) exhibited a linear increase with increasing H, with appreciable net magnetic moments of 3.37 and 3.82 emu/g at room temperature and H = 2 T (Figure 8). These appreciable paramagnetic moments at clinical fields originate from the high spin–orbit magnetic moments of the fast-moving 4f-electrons of Tb^3+^ (^7^F_6_) and Ho^3+^ (^5^I_8_) [48]. Therefore, the nanoparticles can induce strong T_2_ water proton spin relaxations with negligible induction of T_1_ relaxations [53,54], in contrast to Gd^3+^ (s = 7/2), Fe^3+^ (s = 5/2), and Mn^2+^ (s = 5/2) (particularly Gd^3+^), which can induce strong T_1_ and T_2_ water proton spin relaxations owing to their pure electron spin magnetic moments originating from slow moving 4f- or 3d-electrons [28,30,31,32]. Therefore, the magnetic moments of the nanoparticles resulted in negligible r_1_ (i.e., 0.086 and 0.093 s^−1^mM^−1^ for Ln = Tb and Ho, respectively) and appreciable r_2_ (i.e., 3.446 and 3.677 s^−1^mM^−1^ for Ln = Tb and Ho, respectively) values (Figure 10a,b). Under these r_1_ and r_2_ values, the core–shell Ln_2_O_3_@carbon nanoparticles can serve as T_2_ MRI contrast agents because they can exclusively induce T_2_ relaxations while causing minimal T_1_ relaxations, as demonstrated in vitro by negligible and appreciable dose-dependent contrast changes observed in the R_1_ and R_2_ map images, respectively.

As provided in Table 2, the r_1_ and r_2_ values of our nanoparticles are similar to those of carbon-coated Dy_2_O_3_ nanoparticles [12] because of similar spin–orbit electronic magnetic moments of Tb^3+^, Dy^3+^, and Ho^3+^, but are considerably smaller than those of carbon-coated Gd_2_O_3_ nanoparticles [40] because of different (i.e., pure spin) electronic magnetic moments of Gd^3+^ as mentioned above. Compared to free lanthanide metal ions [58] (Table 2), the r_1_ values of our carbon-coated nanoparticles are slightly smaller owing to fewer water coordination numbers in nanoparticles than in free lanthanide metal ions, but our r_2_ values are higher owing to the high density of lanthanide metal ions in the nanoparticles.

## 4. Materials and Methods

### 4.1. Materials

LnCl_3_·6H_2_O (99.9%; Ln = Tb and Ho), D-glucose (>99.5%), NaOH (>99.9%), and triethylene glycol (TEG) (99%) were purchased from Sigma-Aldrich, St. Louis, MO, USA, and used as received. Ethanol (99%) was purchased from Duksan, Ansan, Republic of Korea, and used as received. Triple-distilled water was obtained using a Pure Power I+ system (Human Co., Ltd., Seoul, Republic of Korea). The human prostate carcinoma cell line DU145 and mouse liver normal hepatocyte cell line NCTC 1469, used in this study, were obtained from the American Type Culture Collection (Rockville, MD, USA) and the Korean Cell Line Bank (Seoul, Republic of Korea), respectively.

### 4.2. Synthesis of Ultrasmall Core–Shell Ln_2_O_3_@Carbon Nanoparticles (Ln = Tb and Ho)

**Figure 12:**
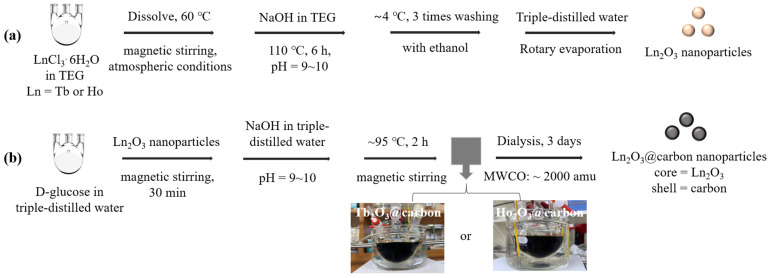
Synthesis of ultrasmall core–shell Ln_2_O_3_@carbon nanoparticles (Ln = Tb and Ho) in two steps: (**a**) synthesis of ultrasmall Ln_2_O_3_ nanoparticles in TEG and (**b**) carbon coating of these nanoparticles using D-glucose as carbon source in aqueous solution.

Ultrasmall core–shell Ln_2_O_3_@carbon nanoparticles (Ln = Tb and Ho) were synthesized in two steps (Figure 12a,b): (i) synthesis of ultrasmall Ln_2_O_3_ nanoparticles in TEG and (ii) carbon coating of the above nanoparticles using D-glucose as a carbon source in an aqueous solution. In the first step, we prepared a precursor solution [made by dissolving 1.0 mmol of LnCl_3_·6H_2_O (Ln = Tb or Ho) in 20 mL of TEG within a 100 mL three-necked round bottom flask] and a solution of 4 mmol of NaOH in 10 mL of TEG contained in a 50 mL beaker. The temperature was controlled by suspending the reaction flask in a silicone oil bath placed on a hot plate. After dissolution of LnCl_3_·6H_2_O in TEG via magnetic stirring at 60 °C under atmospheric conditions, followed by the addition of the NaOH solution until the solution pH reached 9 to 10, the temperature of the mixed solution was slowly raised to 110 °C and maintained for 6 h. After being cooled to room temperature, the solution was diluted with 500 mL of ethanol in a beaker, then magnetically stirred for ~5 min, and kept in a refrigerator (at ~4 °C) until the nanoparticles settled to the beaker bottom. After decanting the top transparent liquid, the remaining Ln_2_O_3_ nanoparticle solution was diluted with ethanol again: this washing process was repeated three times to remove unreacted precursors, NaOH, and TEG. Ethanol was removed from the product solution by diluting it with 100 mL of triple-distilled water and then concentrating it to ~10 mL using a rotary evaporator. In the second step, the above concentrated Ln_2_O_3_ nanoparticle solution was added to a mixture consisting of 1.0 mmol of D-glucose dissolved in 10 mL of triple-distilled water within a 100 mL three-necked round bottom flask; the mixed solution was magnetically stirred for 30 min, followed by the addition of NaOH solution (4 mmol of NaOH in 5 mL of triple-distilled water) until the solution pH reached 9–10. The mixed solution was magnetically stirred at ~95 °C for 2 h to make the solution color black. After being cooled to room temperature, the solution was transferred to a dialysis bag (MWCO ~2000 amu) and dialyzed against triple-distilled water for 3 days with magnetic stirring; outside water was replaced with fresh water three times. Then, half of the solution volume was concentrated to a powder by freeze-drying it in vacuum prior to characterization.

### 4.3. Characterization Experiments

HRTEM (Titan G2 ChemiSTEM CS Probe, FEI, Hillsboro, OR, USA) was used to measure the d values of the ultrasmall core–shell Ln_2_O_3_@carbon nanoparticles (Ln = Tb and Ho) at a 200kV acceleration voltage. The Ln concentration in an aqueous nanoparticle suspension sample was measured using inductively coupled plasma-atomic emission spectrometry (ICP-AES, Optima 7300DV& Avio500, Perkin Elmer, Waltham, MA, USA). A multi-purpose XRD spectrometer (X’PERT PRO MRD, Philips, The Netherlands) with unfiltered CuKα radiation (λ = 0.154184 Å) was used to determine the crystal structure of the power samples before and after TGA. A DLS particle size analyzer (Zetasizer Nano ZS, Malvern, UK) was used to measure the hydrodynamic diameter (a) and zeta potential values of the ultrasmall core–shell Ln_2_O_3_@carbon nanoparticles dispersed in an aqueous solution. An FT-IR absorption spectrometer (Galaxy 7020A, Mattson Instruments, Inc., Madison, WI, USA) was used to analyze the carbon coating on the nanoparticle surfaces by recording the corresponding spectra using KBr pellets of powder samples. To estimate the carbon coating amount on the nanoparticle surface, an SDT-Q600 analyzer (TA Instruments, New Castle, DE, USA) was used to record the TGA curves of powder samples between room temperature and 900 °C under air flow. EA (Flash 2000, ThermoFisher, Waltham, MA, USA) was used to analyze the surface composition (C/H/O) and coating amount using powder samples. A Raman spectrometer (InVia Reflex, Renishaw, West Dundee, IL, USA) was used to record Raman spectra of the carbon coatings using power samples. Finally, vibrating sample magnetometry (VSM, 7407-S, LakeShore, Westerville, OH, USA) was used to measure the magnetic properties of the nanoparticles using powder samples at room temperature.

### 4.4. In Vitro Cellular Cytotoxicity Assay

The in vitro cellular cytotoxicity of the aqueous nanoparticle suspension samples was assessed using a Luminescent Cell Viability Assay (CellTiter-Glo, Promega, Madison, WI, USA) and a luminometer (Victor 3, Perkin Elmer, Waltham, MA, USA) to quantify the adenosine triphosphate (ATP) levels in live cells. DU145 and NCTC1469 cells were seeded onto a 24-well cell culture plate, followed by incubation for 24 h (5 × 10^4^ cell density, 500 μL cells per well, 5% CO_2_, and 37 °C). Each nanoparticle suspension sample (2 μL), prepared at various concentrations (10, 50, 100, 200, and 500 μM Tb or Ho) by dilution of the original concentrated suspension with a sterile phosphate-buffered saline (PBS) solution, was dropped onto the cells, followed by incubation for 48 h. After three cell viability tests, the obtained values were normalized to those of the control (i.e., untreated) cells.

### 4.5. Measurements of Relaxometric Properties

The T_1_ and T_2_ water proton spin relaxation times, along with R_1_ and R_2_ map images, were measured with a 3 T MRI scanner (Signa Advantage 3 T, GE Medical System, Chicago, IL, USA) equipped with a knee coil at 22 °C, using a series of nanoparticle suspension samples (1.0, 0.5, 0.25, 0.125, 0.0625, and 0.0 mM Tb or Ho) prepared by dilution of the original concentrated samples with triple-distilled water. The r_1_ and r_2_ values were estimated from the slopes of 1/T_1_ and 1/T_2_ plots, respectively, versus the Tb or Ho concentration. The T_1_ relaxation times were determined using an inversion recovery method. The Carr–Purcell–Meiboom–Gill pulse sequence for multiple spin-echo measurements was used to obtain the T_2_ relaxation times. Typical imaging parameters for T_1_ (T_2_) relaxation time measurements were as follows: slice thickness = 5 (5) mm, repetition time = 2000 (2000) ms, echo time = 18 (15, 50, 45, …, 480) ms, number of averaging = 1 (1), echo train length = 17 (1), flip angle = 120 (180) degree, matrix size = 320 × 192 (320 × 192), and field of view = 180 × 108 (180 × 108).

## 5. Conclusions

In this study, ultrasmall core–shell Ln_2_O_3_@carbon nanoparticles (Ln = Tb and Ho) were synthesized for the first time and their physicochemical properties, including in vitro cellular cytotoxicities and water proton spin relaxivities, were characterized as follows.

(1) The ultrasmall core–shell Ln_2_O_3_@carbon nanoparticles (Ln = Tb and Ho) showed nearly monodisperse size distributions (d_avg_ = ~3 nm), along with good colloidal stability and low cellular cytotoxicity.
(2) The ultrasmall core–shell Ln_2_O_3_@carbon nanoparticles (Ln = Tb and Ho) also exhibited negligible r_1_ (i.e., 0.086 and 0.093 s^−1^mM^−1^) and appreciable r_2_ (i.e., 3.446 and 3.677 s^−1^mM^−1^) values for Ln = Tb and Ho, respectively, demonstrating their potential to serve as T_2_ MRI contrast agents, particularly at high applied fields.
(3) The carbon-coating shell exhibited photoluminescence at 460 nm, suitable for application in fluorescent imaging probes. Therefore, the present nanoparticles have dual imaging properties, making them suitable for T_2_ MRI and fluorescent imaging applications, highlighting their novelty.

## Figures and Tables

**Table 1:** Summarized physicochemical properties of core–shell Ln_2_O_3_@carbon nanoparticles (Ln = Tb and Ho).

Core Nanoparticle	d_avg_ (nm)	a_avg_ (nm)	ζ (mV)	Carbon Coating Amount (wt.%)		Net M at 2 T (emu/g)	r_1_ (s^−1^mM^−1^)	r_2_ (s^−1^mM^−1^)
				TGA	EA			
Tb_2_O_3_	3.0	19.5	−48.2	57.7	59.5	3.37	0.086	3.446
Ho_2_O_3_	2.9	21.5	−40.2	58.5	61.8	3.82	0.093	3.677

**Table 2:** Comparison of r_1_ and r_2_ values for core–shell Ln_2_O_3_@carbon nanoparticles (Ln = Gd, Tb, Dy, and Ho).

Core Nanoparticle or Free Ion	d_avg_ (nm)	r_1_ (s^−1^mM^−1^)	r_2_ (s^−1^mM^−1^)	Applied Field (T)	Ref
Tb_2_O_3_	3.0	0.086	3.446	3	This study
Ho_2_O_3_	2.9	0.093	3.677	3	This study
Dy_2_O_3_	3.0	0.1	5.7	3	[12]
Gd_2_O_3_	3.1	16.26	24.12	3	[40]
Gd^3+^	-	15.1	17.9	4.7	[58]
Dy^3+^	-	0.652	0.688	4.7	[58]
Ho^3+^	-	0.494	0.530	4.7	[58]

## Data Availability

The original contributions presented in this study are included in the article. Further inquiries can be directed to the corresponding authors.
